# Can dementia be predicted using olfactory identification test in the elderly? A Bayesian network analysis

**DOI:** 10.1002/brb3.1822

**Published:** 2020-08-31

**Authors:** Ding Ding, Xiaoniu Liang, Zhenxu Xiao, Wanqing Wu, Qianhua Zhao, Yang Cao

**Affiliations:** ^1^ Institute of Neurology Huashan Hospital Fudan University Shanghai China; ^2^ National Clinical Research Center for Aging and Medicine Huashan Hospital Fudan University Shanghai China; ^3^ Clinical Epidemiology and Biostatistics, School of Medical Sciences Örebro University Örebro Sweden

**Keywords:** Bayesian network, cohort, dementia, elderly, olfactory function, olfactory identification test, prediction

## Abstract

**Background:**

Previous studies suggest that olfactory dysfunction is associated with cognitive decline or dementia.

**Objective:**

To find a potential association between the olfactory identification (OI) and dementia onset, and build a prediction model for dementia screening in the older population.

**Methods:**

Nine hundred and forty‐seven participants from the Shanghai Aging Study were analyzed. The participants were dementia‐free and completed OI test using the Sniffin’ Sticks Screening Test‐12 at baseline. After an average of 4.9‐year follow‐up, 75 (8%) of the participants were diagnosed with incident dementia. Discrete Bayesian network (DBN) and multivariable logistic regression (MLR) models were used to explore the dependencies of the incident dementia on the baseline demographics, lifestyles, and OI test results.

**Results:**

In DBN analysis, odors of orange, cinnamon, peppermint, and pineapple, combined with age and Mini‐mental State Examination (MMSE), achieved a high predictive ability for incident dementia, with an area under the receiver operating characteristic curve (AUC) larger than 0.8. The odor cinnamon showed the highest AUC of 0.838 (95% CI: 0.731–0.946) and a high accuracy of 0.867. The DBN incorporating age, MMSE, and one odor test had an accuracy (0.760–0.872 vs. 0.835) comparable to that of the MLR model and revealed the dependency between the variables.

**Conclusion:**

The DBN using OI test may have predictive ability comparable to MLR analysis and suggest potential causal relationship for further investigation. Identification of odor cinnamon might be a useful indicator for dementia screening and deserve further investigation.

## Graphical abstract

The Bayesian Network using olfactory identification test showed good predictive ability for predicting dementia, which suggests potential causal pathway for further investigation. Identification of odor cinnamon might be a useful indicator for dementia screening.

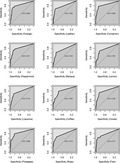

## 1 INTRODUCTION

Dementia is an overall term for diseases and conditions characterized by a decline in memory, language, problem‐solving, and other thinking skills that affect a person's ability to perform everyday activities. Alzheimer's disease (AD) is the most common cause of dementia. There are 47 million people with dementia worldwide. By 2050, the number of people with dementia is estimated to increase to more than 131 million (Prince, Comas‐Herrera, Knapp, Guerchet, & Karagiannidou, 2016). Because effective treatment for dementia is lacking, it is imperative to explore the risk factors and provide early identification of cognitive decline and dementia. Accumulating evidence from both human studies and disease models indicates that intercellular transmission and the subsequent templated amplification of some misfolded proteins (e.g., amyloid‐*β* and *τ*, *α*‐synuclein, and TAR DNA‐binding protein 43) are involved in the onset and progression of various neurodegenerative diseases (Peng, Trojanowski, & Lee, 2020). Except for traditionally recognized mmega‐3 fatty acids, recent findings reveal nicotinamide adenine dinucleotide and related metabolites playing important roles in the adaptation of neurons to a wide range of physiological stressors and in counteracting processes in neurodegenerative diseases, and chronic gamma entrainment and tacrine‐benzofuran hybrids may offer neuroprotective effects, which might provide new therapeutic opportunities (Adaikkan & Tsai, 2020; Fancellu et al., 2020; Lautrup, Sinclair, Mattson, & Fang, 2019). Olfactory dysfunction, which increases substantially with aging, represents an important clinical symptom suggesting the early stage of neurodegenerative disorders (Attems, Walker, & Jellinger, 2015). Previous cross‐sectional and longitudinal population‐based studies suggest that olfactory dysfunction is associated with impairment in various cognitive domains and incident cognitive decline and dementia, and emphasize its essential role as a predictive marker (Roalf et al., 2017; Wehling, Nordin, Espeseth, Reinvang, & Lundervold, 2011).

The Shanghai Aging Study (SAS) is a community‐based cohort study for investigating the progression of cognitive decline in Chinese elderly, with study design, operational procedures, and diagnostic criteria similar to most cohort studies in developed countries and published previously (Ding et al., 2014). At the baseline of SAS, the Sniffin’ Sticks Smell Test‐12 (SSST‐12) was used to examine the olfactory identification (OI) ability of the study participants. Our previous study of the cross‐sectional phase of SAS explored the relation between lower total OI score and mild cognitive impairment (MCI; Liang et al., 2016). At the prospective phase, we further demonstrated that the inability to smell peppermint was associated with a higher risk for incident dementia (Liang et al., 2020). The associations, however, were examined only by multivariable logistic regression (MLR) model. The predictive value of OI test and identification ability of certain odors needs to be further validated.

A Bayesian network (BN) is a probabilistic graphical model that represents a set of variables and their conditional dependencies via a directed acyclic graph (DAG; Scutari & Denis, 2014). BN is ideal for taking an event that occurred and predicting the likelihood that any one of several possible known causes was the contributing factor. Experience has shown that BN and associated methods are geared to reasoning with uncertainty in a way closely resembling physicians (Kammerdiner, Gupal, & Pardalos, 2007; Lucas, 2007; Pearl, 2014). Physicians who address to develop computer‐assisted system for making clinical decisions are frequently confronted by the complexity and uncertainty in the models and prediction. In many cases, the situation is even worse as many of the processes in medicine are only partly known (Lucas, 2007). During the past decade, BN has become an important tool for building decision‐support systems in medical sciences and is now steadily becoming main stream in some areas (Mani, Valtorta, & McDermott, 2005).

Although the BN model has been used in studying gene expression levels for the PD (Mestizo‐Gutiérrez, Jácome‐Delgado, Rosales‐Morales, Cruz‐Ramírez, & Aranda‐Abreu, 2019), and in predicting the AD using clinical data (Khanna et al., 2018), according to our literature search, there is no study that has used the method for predicting dementia using the OI data from observational studies. In this study, by using the data from SAS, we examined the performance of the BN analysis in predicting incident dementia and compared the BN with the MLR model. Our study also aimed to find associations between the baseline variables, including olfactory function, and dementia onset, and to build a prediction model with high performance for dementia screening in the older population. The identification of one or several odors that are sensitive for dementia prediction would benefit large scale population screen programs for dementia prevention and intervention in elders.

## 2 MATERIAL AND METHODS

### 2.1 Study participants

**Figure 1:**
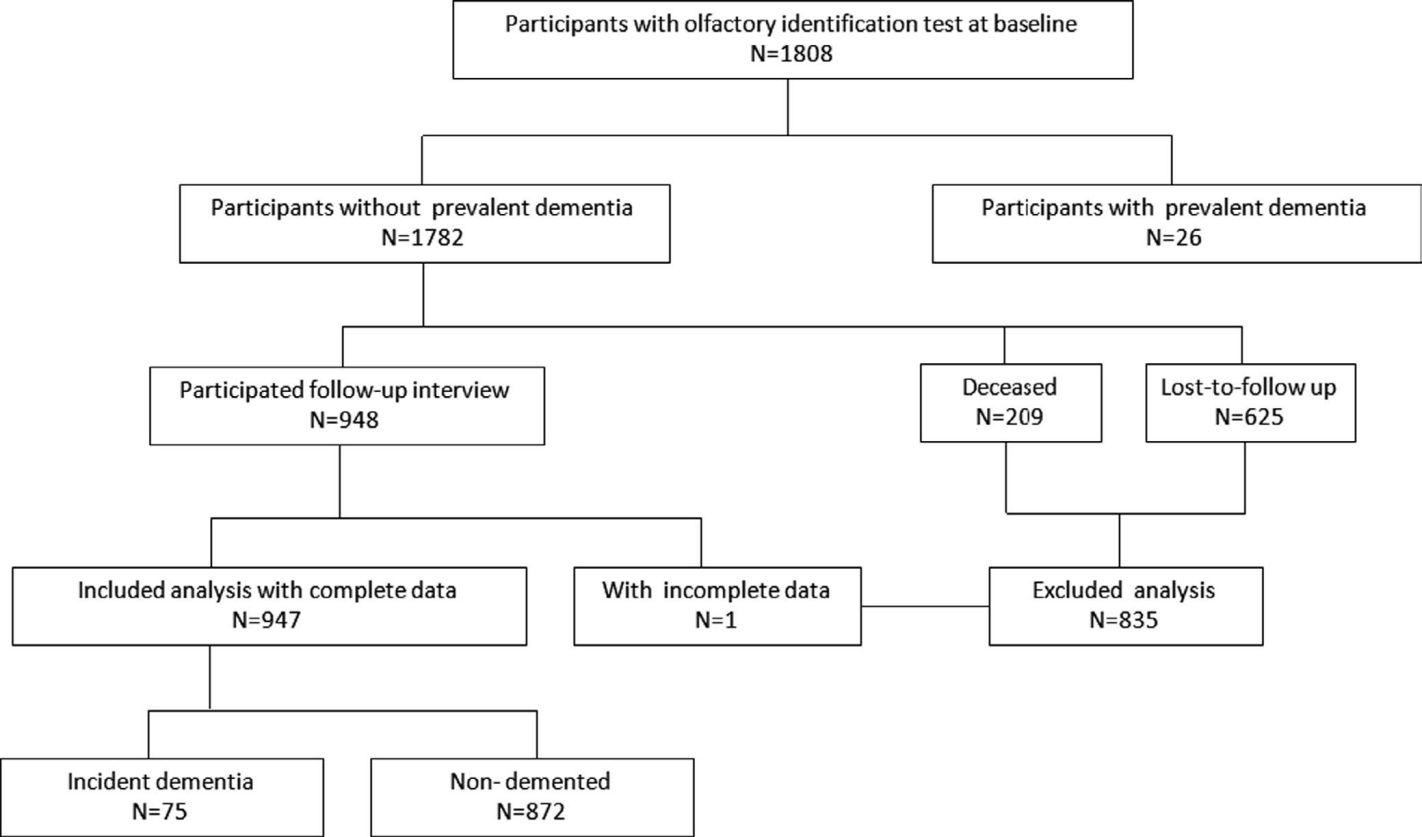
Flow chart of the subcohort participants in the current study

The participants of the current study were a subcohort of SAS. In brief, SAS was designed to establish a prospective community‐based cohort to examine the prevalence and incidence of dementia and MCI in Chinese older adults residing in central Shanghai (Ding et al., 2014). Between January, 2010 and December, 2011, 3,141 participants aged 60 years or older were recruited and completed the clinical interview as the baseline. Among them, 1,782 were assessed for olfactory function by the OI test as the additional examination of the interview. Participants who were diagnosed as dementia‐free at the baseline were contacted between April, 2014 and December, 2016 for a follow‐up interview to determine the new‐onset dementia cases. Participants were excluded if they (a) resided in nursing homes or other institutions; (b) had mental retardation or severe schizophrenia; (c) had severe hearing, vision, or verbal impairment; (d) were undergoing maxillofacial surgery; (e) had chronic obstructive pulmonary disease or had experienced an acute upper respiratory tract infection within 1 week; (f) used alcohol and drugs excessively; (g) had dementia or other severe neurological diseases; or (h) refused to participate, were lost to follow‐up, or were deceased; (i) did not cooperate for a completed data collection at the follow‐up interview. Finally, 947 participants completed the follow‐up interview and were included in the current study (Figure 1). Detailed recruitment and follow‐up procedures of SAS were reported previously (Ding et al., 2014; Liang et al., 2016, 2020).

### 2.2 Baseline data

At the baseline, data on demographics, lifestyles, and medical history of each participant were obtained from a face‐to‐face interview by trained research nurses and neurologists. Height and weight were measured and used to calculate the body mass index (BMI). History of chronic diseases, including hypertension, coronary artery disease (CAD), diabetes, and stroke were asked and confirmed by medical records maintained by participants (Ding et al., 2014). Depression was defined as present if the scores of Center for Epidemiologic Studies Depression Scale (CESD) ≥16 (Eaton, Smith, Ybarra, Muntaner, & Tien, 2004).

OI was assessed by using the Sniffin’ Sticks Screening Test‐12 (SSST‐12), which consists of 12 odors (orange, leather, cinnamon, peppermint, banana, lemon, liquorice, coffee, cloves, pineapple, rose, and fish) presenting on felt‐tip sticks (Wolfensberger, 2000). The SSST‐12 kit was produced by Burghart Medical Technology, Hamburg, Germany (Burghart Medical Technology). Participants were asked to sniff each odor sticks and to choose one of four answers from a list that described best the odor. The administration methods of SSST‐12 were described in detail elsewhere (Liang et al., 2016).

DNA was extracted from blood or saliva collected from the participants at the baseline (Ding et al., 2015). Apolipoprotein (APOE) genotyping was conducted using the TaqMan SNP method (Smirnov, Morley, Shin, Spielman, & Cheung, 2009). The presence of at least one *ε*4 allele was defined as APOE‐*ε*4 allele positive.

### 2.3 Diagnosis of cognitive function

Both at the baseline and follow‐up, the cognitive function of each participant was evaluated by a battery of neuropsychological tests: (1) Mini‐mental State Examination (MMSE); (2) Conflicting Instructions Task (Go/No Go Task); (3) Stick Test; (4) Modified Common Objects Sorting Test; (5) Auditory Verbal Learning Test; (6) Modified Fuld Object Memory Evaluation; (7) Trail‐making test A&B; and (8) Ren Ming Bi (RMB, Chinese currency) test. The neuropsychological tests were administered by study psychometrists according to the education level of each participant. Participants with at least 6 years of education were administered by tests (1) to (5) and (7), and those with less than 6 years of education were administered by tests (1) to (4) and (6) and (8). The normative data and detailed description of these tests were reported elsewhere (Ding et al., 2014, 2015).

Two study neurologists, one neuropsychologist and one neuroepidemiologist reviewed the functional, medical, neurological, psychiatric, neuropsychological data, and Clinical Dementia Rating (CDR) and Activity of Daily Living (ADL) scale of the participants and reached a consensus diagnosis regarding the presence of dementia according to the Diagnostic and Statistical Manual of Mental Disorders IV (DSM–IV) criteria (American Psychiatric Association, 1994; Ding et al., 2014, 2015).

### 2.4 Descriptive analysis

Data of demographics, lifestyle, medical history, and OI test results were presented as mean with standard deviation (*SD*) or median with interquartile range (IQR) for continuous variable, and as number and percentage for categorical variables. Difference between groups was tested by the chi‐squared test for categorical variables, and Student's *t* test or Mann–Whitney U test for continuous variables. A two‐sided *p*‐value <.05 was considered as statistically significant. The descriptive analyses were performed using Stata 16.0 (StataCorp LLC).

### 2.5 Bayesian network analysis

Prediction for incident dementia was conducted using multinomial discrete BN (DBN). Before entering the DBN, continuous variables were discretized into ten categories based on their own deciles. Although at the cost of losing some information, the discretization may accommodate skewness of the variables and nonlinear relationships between them, and speed up the computation substantially (Hartemink, 2001; Sachs, Perez, Pe'er, Lauffenburger, & Nolan, 2005; Scutari & Denis, 2014).

The K‐fold cross‐validation method was used during the DBN structure learning and validation. K‐fold cross‐validation is a standard way to obtain unbiased estimates of a model's goodness of fit and to handle the overfitting problem when applying only one single dataset in statistical learning (James, Witten, Hastie, & Tibshirani, 2013). In the current study, we randomly split the dataset into five equal partitions, instantiated five identical DBNs, and trained each one on four partitions while validating on the remaining partition. In each iteration, the prediction was made for the one held‐out partition. In the end, the validation for the whole dataset was obtained by combining the prediction for the five held‐out partitions (James et al., 2013). When learning the structure of the DBNs, an initial black list was used to block the arcs from dementia to the baseline variables, and the arcs from the other baseline variables to sex and age, and no other constraints were used. The hill‐climbing (HC) algorithm was used to learn the structure of the DBNs. The HC starts from a network with no arcs, then adds, removes, and reverses one arc at a time, and finally picks the change that increases the network's Bayesian information criterion score the most (Scutari & Denis, 2014).

The performance of the DBNs was evaluated using metrics including sensitivity, specificity, accuracy, and area under the receiver operating characteristic (ROC) curve. Terminology and derivations of the metrics were given in detail elsewhere (Cao, Fang, Ottosson, Naslund, & Stenberg, 2019). A successful prediction model for incident dementia was defined as one with an area under the ROC curve (AUC) >0.7 (Mandrekar, 2010; Marzban, 2004).

We also compared the performance of the DBNs with that of the traditional stepwise MLR model based on bidirectional variable selection. The K‐fold cross‐validation was also used for the stepwise MLR analysis.

The DBNs were constructed using the package *bnlearn* in software R version 3.62 (R Foundation for Statistical Computing) (Scutari & Denis, 2014). The stepwise MLR analysis was conducted using package *MASS* in R (Ripley, 2002), and a two‐sided *p*‐value <.05 was considered as statistically significant.

## 3 RESULTS

### 3.1 Characteristics of the study participants at baseline

In general, there was no significant difference in the baseline characteristics between the 835 excluded participants and the 947 included participants, except for the percentage of positive APOE‐*ε*4 carriers (18.2% vs. 16.5%, *p* < .001). Although the mean age and BMI of the included participants were a bit larger (70.51 vs. 70.12 years, and 24.49 vs. 24.07 kg/m^2^, respectively), the differences were not clinically significant (Table 1).

**Table 1:** Characteristics of the study participants at baseline

Variable	Total	Excluded	Included	*p*‐value
*N*	1,782	835	947	
Male, *n* (%)	818 (45.9)	382 (45.7)	436 (46.0)	.940
Age (year),mean (*SD*)	70.12 (7.1)	69.67 (7.5)	70.51 (6.8)	.014
BMI (kg/m^2^), mean (*SD*)	24.29 (3.7)	24.07 (3.3)	24.49 (3.9)	.016
Height (m),mean (*SD*)	161.87 (8.9)	162.26 (8.7	161.53 (9.0)	.084
Weight (kg),mean (*SD*)	63.78 (11.3)	63.53 (11.00)	63.99 (11.6)	.385
Education (year), median [IQR]	12.0 [9.0, 15.0]	12.0 [9.0, 15.0]	12.0 [10.0, 15.0]	.407
Smoking, *n* (%)	185 (10.4)	84 (10.1)	101 (10.7)	.734
Drinking, *n* (%)	142 (8.0)	55 (6.6)	87 (9.2)	.053
CAD, *n* (%)	194 (10.9)	103 (12.3)	91 (9.6)	.077
Hypertension, *n* (%)	962 (54.0)	463 (55.4)	499 (52.7)	.264
Diabetes, *n* (%)	247 (13.9)	124 (14.9)	123 (13.0)	.286
Depression, *n* (%)	277 (15.5)	141 (16.9)	136 (14.4)	.161
Stroke, *n* (%)	202 (11.3)	91 (10.9)	111 (11.7)	.637
APOE‐*ε*4positive, *n* (%)	308 (17.3)	152 (18.2)	156 (16.5)	<.001
Correct answer to odors in the OI test				
Orange, *n* (%)	1,365 (76.6)	623 (74.6)	742 (78.4)	.071
Leather, *n* (%)	1,002 (56.2)	468 (56.0)	534 (56.4)	.923
Cinnamon, *n* (%)	770 (43.2)	353 (42.3)	417 (44.0)	.484
Peppermint, *n* (%)	1,622 (91.0)	760 (91.0)	862 (91.0)	1.000
Banana, *n* (%)	1,164 (65.3)	555 (66.5)	609 (64.3)	.365
Lemon, *n* (%)	960 (53.9)	452 (54.1)	508 (53.6)	.874
Liquorice, *n* (%)	957 (53.7)	450 (53.9)	507 (53.5)	.919
Coffee, *n* (%)	1,642 (92.1)	773 (92.6)	869 (91.8)	.584
Cloves, *n* (%)	919 (51.6)	422 (50.5)	497 (52.5)	.441
Pineapple, *n* (%)	1,234 (69.2)	579 (69.3)	655 (69.2)	.977
Rose, *n* (%)	1,112 (62.4)	509 (61.0)	603 (63.7)	.257
Fish, *n* (%)	1,475 (82.8)	705 (84.4)	770 (81.3)	.093
OIS, median [IQR]	8.0 [7.0, 9.0]	8.0 [7.0, 9.0]	8.0 [7.0, 10.0]	.420
MMSE, median [IQR]	29.0 [28.0, 30.0]	29.0 [27.0, 30.0]	29.0 [28.0, 30.0]	.241

Abbreviations: APOE, apolipoprotein; BMI, body mass index; CAD, coronary artery disease; IQR, interquartile range; MMSE, Mini‐mental State Examination score; OIS, olfactory identification sum score; *SD*, standard deviation.

After a mean of 4.9 (*SD* = 0.8) years follow‐up, 75 of the 947 included participants were diagnosed with new‐onset dementia. Compared to the 872 participants without dementia, the 75 dementia cases were 8‐year older (77.8 vs. 69.9 years) when recruited. Although there was no statistically significant difference in baseline BMI between the dementia cases and those without dementia (controls) (24.5 vs. 24.5 kg/m^2^), the cases averagely were shorter (156.5 vs. 162.0 cm), weighed less (59.1 vs. 64.4 kg), and had less education (9 vs. 12 years). CAD, stroke, and APOE‐*ε*4 positive were more frequently observed in the cases (Table 2). The participants with incident dementia had lower correct identification rate in eight odors (leather, cinnamon, peppermint, banana, liquorice, coffee, rose, fish) among the 12 ones in the baseline OI test (Table 2). The cases were also showed lower OI sum score (OIS) and MMSE score at baseline (Table 2).

**Table 2:** Characteristics of dementia cases and controls at baseline (*N* = 947)

Variable	Controls (*n* = 872)	Incident dementia cases (*n* = 75)	*p*‐value
Male, *n* (%)	407 (46.7)	29 (38.7)	.225
Age (year), mean (*SD*)	69.9 (6.5)	77.8 (5.6)	<.001
BMI (kg/m^2^), mean (*SD*)	24.5 (3.5)	24.5 (7.0)	.988
Height (cm), mean (*SD*)	162.0 (8.4)	156.5 (13.2)	<.001
Weight (kg), mean (*SD*)	64.4 (11.5)	59.1 (12.2)	<.001
Education (year), median [IQR]	12.0 [12.0, 15.0]	9.0 [6.0, 12.5]	<.001
Smoking, *n* (%)	93 (10.7)	8 (10.7)	1.000
Drinking, *n* (%)	83 (9.5)	4 (5.3)	.319
CAD, *n* (%)	78 (8.9)	13 (17.3)	.031
Hypertension, *n* (%)	451 (51.7)	48 (64.0)	.054
Diabetes, *n* (%)	113 (13.0)	10 (13.3)	1.000
Depression, *n* (%)	120 (13.8)	16 (21.3)	.105
Stroke, *n* (%)	92 (10.6)	19 (25.3)	<.001
APOE‐*ε*4positive, *n* (%)	137 (15.7)	19 (25.3)	.046
Correct answer to odors in the OI test			
Orange, *n* (%)	687 (78.8)	55 (73.3)	.340
Leather, *n* (%)	504 (57.8)	30 (40.0)	.004
Cinnamon, *n* (%)	397 (45.5)	20 (26.7)	.002
Peppermint, *n* (%)	808 (92.7)	54 (72.0)	<.001
Banana, *n* (%)	578 (66.3)	31 (41.3)	<.001
Lemon, *n* (%)	468 (53.7)	40 (53.3)	1.000
Liquorice, *n* (%)	478 (54.8)	29 (38.7)	.010
Coffee, *n* (%)	809 (92.8)	60 (80.0)	<.001
Cloves, *n* (%)	465 (53.3)	32 (42.7)	.098
Pineapple, *n* (%)	603 (69.2)	52 (69.3)	1.000
Rose, *n* (%)	570 (65.4)	33 (44.0)	<.001
Fish, *n* (%)	717 (82.2)	53 (70.7)	.021
OIS, median [IQR]	8.0 [7.0, 10.0]	7.0 [5.0, 8.0]	<.001
MMSE, median [IQR]	29.0 [28.0, 30.0]	27.0 [25.0, 28.5]	<.001

Abbreviations: APOE, apolipoprotein; BMI, body mass index; CAD, coronary artery disease; IQR, interquartile range; MMSE, Mini‐mental State Examination score; OIS, olfactory identification sum score; *SD*, standard deviation.

### 3.2 Structure of the Bayesian networks and their performance

When no other constraints except for the initial black list were adopted in the DBN structure learning process using the HC algorithm, the probability of dementia incidence was found only dependent on age (Figure S1). Although there were dependencies observed among demographic variables and among odors, separately, no dependency was observed between the two groups of the variables. Besides, no dependency was observed for education, MMSE, and APOE‐*ε*4.

When using the initial DBN to predict the incident dementia, that is, only age used as a predictor, the accuracy of the model is 0.756 and 0.619 in the training and validation, respectively. And the corresponding AUCs are 0.836 (95% confidence interval (CI): 0.794–0.879) and 0.772 (95% CI: 0.653–0.891) (Table 3).

**Table 3:** Performance of the predictive models

	Training						Validation					
	Sensitivity	Specificity	Accuracy	AUC	95% CI of AUC		Sensitivity	Specificity	Accuracy	AUC	95% CI of AUC	
					Lower	Upper					Lower	Upper
Initial DBN^a^	0.786	0.754	0.756	0.836	0.794	0.879	0.842	0.594	0.619	0.772	0.653	0.891
Logistic^b^	0.907	0.798	0.807	0.915	0.884	0.946	0.880	0.831	0.835	0.914	0.879	0.944
DBN‐logistic^c^	1.000	0.994	0.995	1.000	1.000	1.000	0.947	0.123	0.212	0.559	0.486	0.633
DBN including^d^												
Age+Orange	0.786	0.754	0.756	0.845	0.803	0.886	0.737	0.712	0.714	0.730	0.606	0.854
Age+Leather	0.875	0.708	0.720	0.853	0.815	0.890	0.737	0.700	0.704	0.751	0.623	0.880
Age+Cinnamon	0.839	0.726	0.735	0.856	0.818	0.893	0.895	0.600	0.630	0.779	0.672	0.886
Age+Peppermint	0.821	0.738	0.744	0.864	0.825	0.903	0.737	0.676	0.683	0.736	0.608	0.863
Age+Banana	0.893	0.674	0.690	0.852	0.812	0.892	0.737	0.759	0.757	0.771	0.650	0.893
Age+Lemon	0.839	0.711	0.720	0.846	0.807	0.885	0.737	0.688	0.693	0.753	0.636	0.870
Age+Liquorice	0.839	0.717	0.726	0.851	0.813	0.889	0.684	0.735	0.730	0.766	0.649	0.883
Age+Coffee	0.839	0.738	0.745	0.850	0.809	0.891	0.842	0.594	0.619	0.766	0.645	0.887
Age+Cloves	0.839	0.707	0.716	0.843	0.802	0.884	0.842	0.635	0.656	0.773	0.655	0.891
Age+Pineapple	0.786	0.754	0.756	0.844	0.803	0.885	0.737	0.729	0.730	0.750	0.637	0.864
Age+Rose	0.893	0.677	0.693	0.854	0.815	0.893	0.737	0.718	0.720	0.767	0.643	0.890
Age+Fish	0.857	0.704	0.715	0.853	0.816	0.891	0.789	0.671	0.683	0.772	0.660	0.885
Age+MMSE+Orange	1.000	0.808	0.822	0.947	0.929	0.964	0.722	0.888	0.872	0.826	0.715	0.937
Age+MMSE+Leather	1.000	0.839	0.851	0.953	0.937	0.968	0.684	0.873	0.854	0.783	0.664	0.902
Age+MMSE+Cinnamon	1.000	0.833	0.846	0.952	0.936	0.968	0.778	0.877	0.867	0.838	0.731	0.946
Age+MMSE+Peppermint	1.000	0.805	0.819	0.951	0.934	0.968	0.722	0.865	0.851	0.817	0.703	0.932
Age+MMSE+Banana	1.000	0.853	0.864	0.958	0.944	0.972	0.632	0.862	0.839	0.743	0.623	0.863
Age+MMSE+Lemon	1.000	0.840	0.852	0.951	0.935	0.967	0.684	0.837	0.822	0.767	0.652	0.881
Age+MMSE+Liquorice	1.000	0.843	0.855	0.951	0.935	0.967	0.684	0.844	0.828	0.774	0.657	0.890
Age+MMSE+Coffee	1.000	0.789	0.805	0.949	0.931	0.967	0.789	0.756	0.760	0.793	0.683	0.903
Age+MMSE+Cloves	1.000	0.833	0.846	0.956	0.940	0.971	0.737	0.830	0.821	0.792	0.679	0.905
Age+MMSE+Pineapple	1.000	0.832	0.844	0.950	0.934	0.967	0.789	0.825	0.822	0.836	0.730	0.942
Age+MMSE+Rose	1.000	0.848	0.859	0.952	0.937	0.968	0.667	0.845	0.827	0.775	0.649	0.901
Age+MMSE+Fish	1.000	0.849	0.860	0.959	0.945	0.973	0.632	0.904	0.876	0.777	0.655	0.899

^a^ Incident dementia was only dependent on age.

^b^ Multivariable logistic regression model using the variables based on the bidirectional stepwise selection.

^c^ Incident dementia was dependent on the variables used in the multivariable logistic regression.

^d^ Discrete Bayesian networks (DBN) including dependency of incident dementia on the variables below.

The bidirectional stepwise MLR analysis indicated that baseline age, education, APOE‐*ε*4, peppermint, banana, pineapple, and MMSE were statistically significantly associated with incident dementia (Table S1). The accuracy of the MLR model for predicting the incident dementia is 0.807 and 0.835 in the training and validation, respectively, and the corresponding AUCs are 0.915 (95% CI: 0.884–0.946) and 0.914 (95% CI: 0.879–0.944) (Table 3). However, the DBN including the dependency of incident dementia on all the variables selected by the stepwise MLR analysis performed much worse than the initial DBN did, with an accuracy of 0.212 and an AUC of 0.559 (95% CI: 0.486–0.633) (Table 3).

To investigate whether including simple dependencies of incident dementia on OI test and other baseline variables may improve the performance of the initial DBN, in addition to age, we included the arcs from a single odor to dementia (i.e., the dependencies of incident dementia on a single odor) into the DBN one by one first. It turned out that cinnamon performed best for prediction in validation, with an AUC of 0.779 (95% CI: 0.672–0.886). Although the accuracy (0.630) of the DBN is relative low because of the low specificity (0.600), its sensitivity is as high as 0.895 (Table 3).

**Figure 2:**
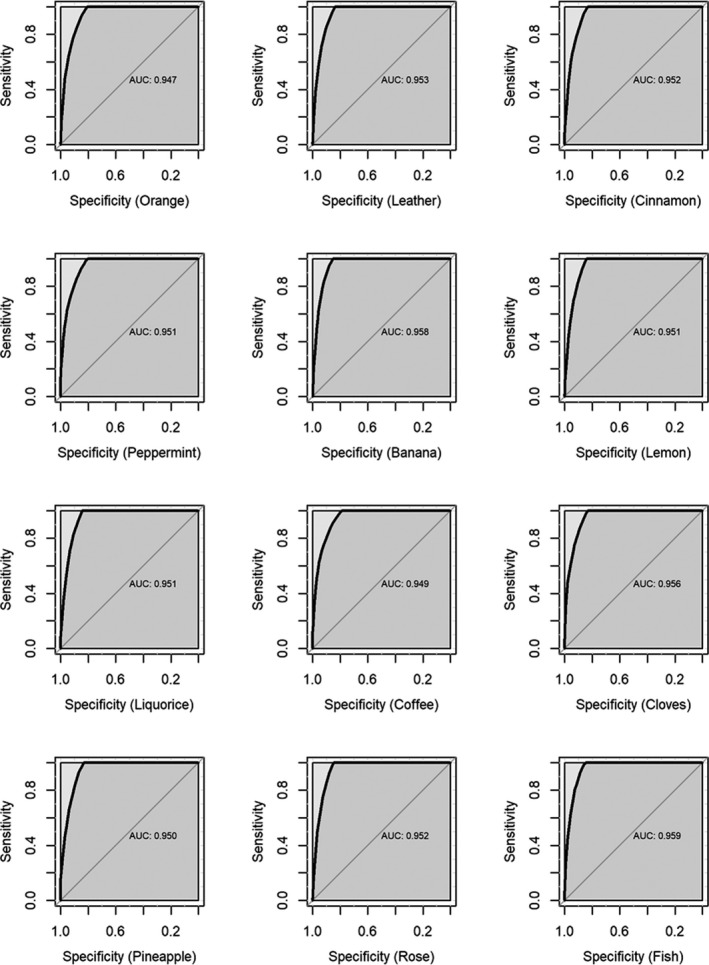
ROCs of the discrete Bayesian networks including dependency of incident dementia on baseline age, MMSE, and one single odor in the training

**Figure 3:**
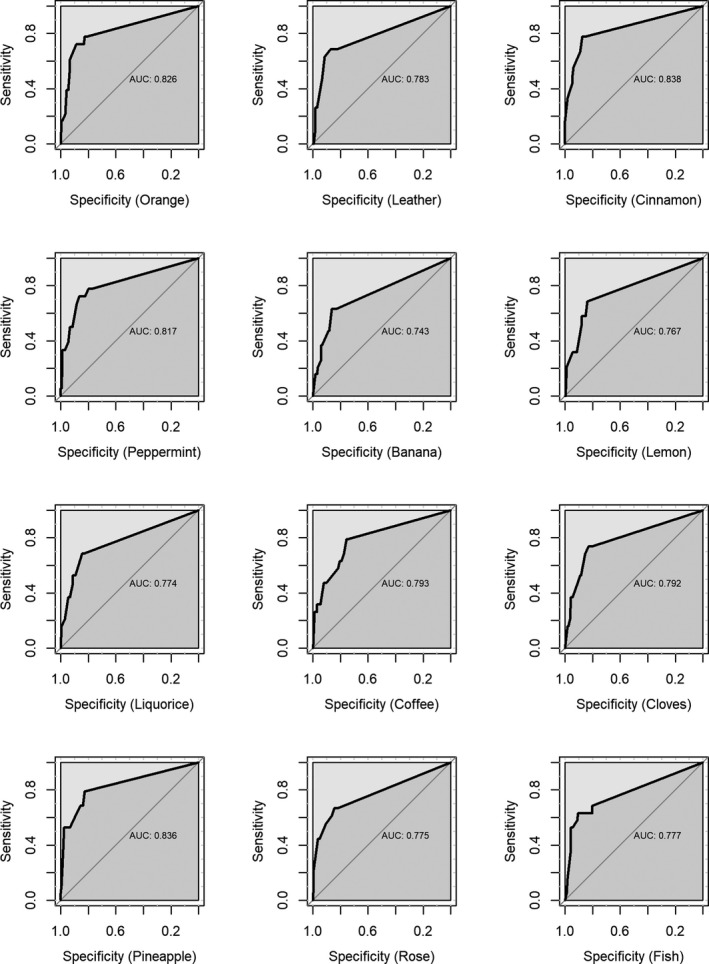
ROCs of the discrete Bayesian networks including dependency of incident dementia on baseline age, MMSE, and one single odor in the validation

To further improve predictive ability of the DBN, we incorporated other statistically significant variables of the MLR analysis in the DBN one by one. It turned that the DBNs incorporating dependencies on a single odor and MMSE performed best in validation. The performance metrics of the DBNs including the dependencies on age, MMSE, and one single odor are shown in Table 3, and the ROCs are shown in Figures 2 and 3 for the training and validation, respectively. In general, using baseline age, MMSE and one odor among orange, cinnamon, peppermint, and pineapple may achieve a very good prediction in validation (AUC > 0.80) (Table 3 and Figure 3).

**Figure 4:**
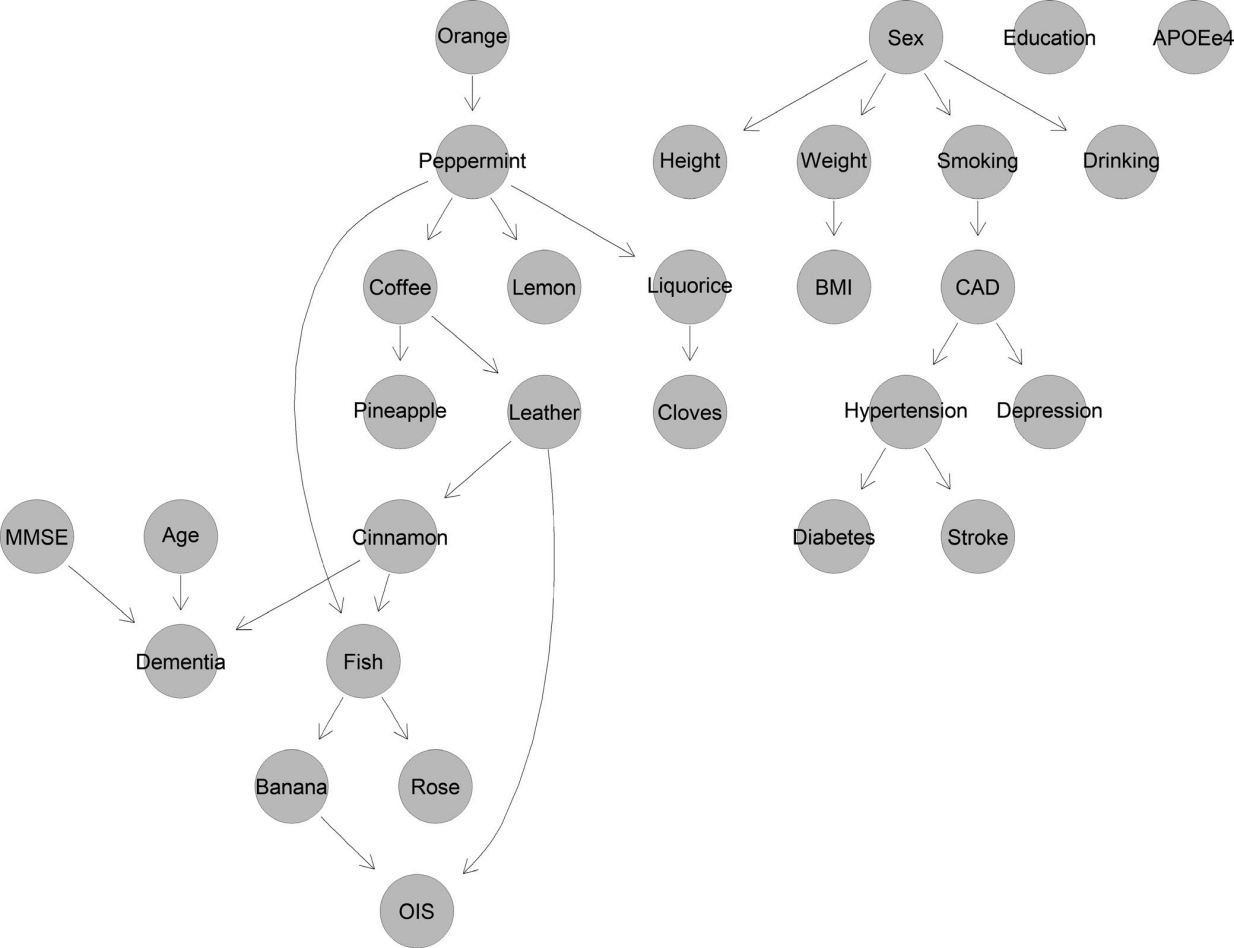
Structure of the discrete Bayesian network (DBN) including the dependency of incident dementia on baseline age, MMSE, and cinnamon

Again, the DBN including the dependency of dementia incidence on cinnamon showed the highest AUC of 0.838 (95% CI: 0.731–0.946) and a high accuracy (0.867) (Table 3). The structure of the DBN is shown in Figure 4.

Performance of the DBNs including the dependency of incident dementia on other baseline variables is shown in Table S2. Compared to the DBNs including the dependencies of incident dementia on age, MMSE, and one odor, DBNs including more dependencies of incident dementia showed worse predictive ability (Table 3 and Table S2).

## 4 DISCUSSION

The DBN analysis in our study indicated that using baseline age, MMSE, and one odor among orange, cinnamon, peppermint, and pineapple may achieve a very good prediction (AUC > 0.80) for incident dementia. Cinnamon odor is an indicator with a high sensitivity of 0.895.

Although the underlying mechanism is not ascertain, olfactory dysfunction is known as one of the early symptoms of some neurodegenerative disorders, such as AD and Parkinson's disease (PD) (Hawkes, Shephard, & Daniel, 1997; Koss, 1986; Serby, Corwin, Conrad, & Rotrosen, 1985). This may provide a perspective into the process of early anatomical change in neurodegenerative disease. Some evidence indicated that AD‐related pathology would first occur in the olfactory bulbs and tracts, where amyloid‐β protein (Aβ), tau, and α‐synuclein are concentrated (Schofield, Finnie, & Yong, 2014). The lesion involves multiple levels of the olfactory system as it progresses, including the surrounding olfactory bulb, olfactory epithelium, and olfactory pathways connecting cognitive regions in the brain (Attems, Walker, & Jellinger, 2014). A meta‐analysis provided evidence that in AD higher order olfactory functions appear to be more strongly affected than in PD. The stronger deficits found in odor identification and recognition in AD may thus be interpreted as the sum of perceptual and cognitive deficits, whereas detection thresholds deficits in PD, might be less dependent on cognition (Rahayel, Frasnelli, & Joubert, 2012). Braak et al indicated that AD pathology in the olfactory system happens during as “transentorhinal stage” and “limbic stage” and involves central olfactory regions such as entorhinal and piriform cortices more than the bulb (Braak et al., 1996). This is also possibly a reason why AD patients are more impaired in cognitively demanding tests of olfaction (such as identification) compared to sensory tests (such as threshold).

Many studies have examined the use of olfactory identification test as a predictor of the development of dementia (Roberts et al., 2016). Combining early markers such as MMSE, APOE genotype, and olfactory identification deficit have been shown strong prediction capability for dementia in long‐term cohort studies, however, the prediction models were mainly based on logistic regression analysis, and the performance of the models was not validated using unseen data or cross‐validation (Conti et al., 2013; Devanand et al., 2008, 2015; Liang et al., 2020; Stanciu et al., 2014). Sun et al. summarized the findings of two prospective longitudinal cohort studies and 30 cross‐sectional studies, and concluded that although a positive association between poorer performance on olfactory and dementia was demonstrated, hyposmia had only moderately predictive value (Sun, Raji, MacEachern, & Burke, 2012).

There are several advantages of using BN. First, commonly used methods in epidemiological studies such as logistic regression and related methods do not take account of conditional dependence that may exist between the covariates. Conditional dependence between some of the risk factors may be already known or may be regarded as plausible on biological grounds (Karhausen, 1987; Susser, 1991). However, such information could be incorporated into BN models to reveal the potential relationships between the health or disease status and the associated risk factors (Li, Shi, & Satz, 2008). Second, high correlation among predictors has long been an annoyance in regression analysis. The crux of the problem is that the linear regression model assumes each predictor has an independent effect on the response that can be encapsulated in the predictor's regression coefficient. As opposed to creating problems of multicollinearity, the associations between candidate predictor variables are naturally accounted for when defining a BN’s conditional probability distributions using. The HC algorithm used in the study may search a structure starting from either an empty, full, or possibly random DAG, or an initial DAG chosen according to existing knowledge. The main loop then consists of attempting every possible single‐edge addition, removal, or reversal relative to the current candidate network. The change that increases the score the most then becomes the next candidate. The process iterates until a change in a single‐edge no longer increases the score. By gradually taking into account of the relationships between the variables, the problem of multicollinearity therefore can be reduced in a BN analysis (Nguefack‐Tsague, 2011). Third, the DAG proposed by the BN captures the dependence structure of multiple variables and, used appropriately, allows more robust conclusions about the direction of causation. BN analysis revealed a richer structure of relationships than could be inferred using the traditional multivariable regression methods such as logistic regression and highlight potential pathway unseen previously for further investigation (Moffa et al., 2017).

However, there are also some limitations in our study. We collected data on potential confounders as many as possible to be used in the analysis model. But there are still uncollected confounders, such as occupation, leisure time activities, which could influence the cognitive function. APOE has been identified as a major genetic risk factor for AD. However, the APOE frequencies have a significant variation in populations with different ethnicities. The frequency of the APOE‐*ε*4 allele in our Shanghai Aging Study is 9.3%, which is in the range of that in Asian populations (6.3%–9.3%), but lower than that in Caucasian and African American populations (11%–27%) (Ding et al., 2014). In our study, the APOE‐*ε*4 allele did not link to any of the parameters due to the relatively small sample size and the low frequency of the APOE‐*ε*4 allele in our study population. Further studies with larger sample size may have the condition to explore if the APOE‐*ε*4 allele is independent or has synergistic effects with other risk factors for cognitive impairment. The relatively high lost to follow‐up rate in the prospective phase of data collection may induce selection bias, although most characteristics of participants followed or lost to followed are similar. Our dataset includes both continuous and binary variables. To reduce the complexity of the networks and computing time, we discretized the continuous variables for the DBN analysis, which may result in information reduction in the analyses. A better solution would be a hybrid BN with use of Markov chain Monte Carlo techniques (Scutari & Denis, 2014). We also noticed that including too many dependencies of incident dementia on the potential baseline predictors only incorporates noise rather than information in prediction, which results in a very low performance in validation (accuracy = 0.212 and AUC = 0.559) and suggests the overfitting problem in the DBN. Although limited by the software packages currently available and adopting the compromising methods so far, we would like to explore the hybrid BN in the future and see whether it could improve the predictive ability further. In the SSST‐12 test, the order of the identification items might also contribute to the effect. However, we could hardly find a reference that explained if the item order is randomized or not. Additionally, the result on each item might be affected by an unknown interaction between the odor and the response options, and this should be carefully considered in future studies.

## 5 CONCLUSION

The DBN incorporating age, MMSE, and one odor test may have predictive ability comparable to MLR analysis, while DBN may also reveal the dependency between the variables in static data and suggest potential causal relationship for further investigation.

## CONFLICT OF INTEREST

The authors declare that there is no conflict of interest related to the paper.

## AUTHOR CONTRIBUTIONS

Conceptualization, Ding Ding and Yang CAO; Data curation, Xiaoniu Liang, Zhenxu Xiao, Wanqing Wu, and Qianhua Zhao; Formal analysis, Ding Ding and Yang CAO; Funding acquisition, Ding Ding; Investigation, Ding Ding, Xiaoniu Liang, Zhenxu Xiao, Wanqing Wu, and Qianhua Zhao; Methodology, Yang CAO; Project administration, Ding Ding; Software, Yang CAO; Validation, Ding Ding and Yang CAO; Visualization, Yang CAO; Writing – original draft, Ding Ding and Yang CAO; Writing – review & editing, Ding Ding, Xiaoniu Liang, Zhenxu Xiao, Wanqing Wu, Qianhua Zhao, and Yang CAO.

## ETHICAL APPROVAL

The study is an observational study and was approved by the Medical Ethical Committee of Huashan Hospital, Fudan University, Shanghai, China (approval number: 2009‐195). All participants and/or their legal guardians gave their written informed consent for participation in the study. There is no personal identification disclosed in our data.

### Peer Review

The peer review history for this article is available at https://publons.com/publon/10.1002/brb3.1822.

## Supporting information

Supinfo

## Data Availability

The data are not publicly available but may be available upon reasonable request and with permission of the principle investigator Dr. Ding Ding (dingding@huashan.org.cn).

## References

[brb31822-bib-0001] Adaikkan, C. , & Tsai, L. H. (2020). Gamma entrainment: Impact on neurocircuits, glia, and therapeutic opportunities. Trends in Neurosciences, 43(1), 24–41. 10.1016/j.tins.2019.11.001 31836315

[brb31822-bib-0002] American Psychiatric Association (1994). DSM‐IV: Diagnostic and statistical manual of mental disorders. Washington, DC: American Psychiatric Press Inc.

[brb31822-bib-0003] Attems, J. , Walker, L. , & Jellinger, K. A. (2014). Olfactory bulb involvement in neurodegenerative diseases. Acta Neuropathologica, 127(4), 459–475. 10.1007/s00401-014-1261-7 24554308

[brb31822-bib-0004] Attems, J. , Walker, L. , & Jellinger, K. A. (2015). Olfaction and aging: A mini‐review. Gerontology, 61(6), 485–490. 10.1159/000381619 25968962

[brb31822-bib-0005] Braak, H. , Braak, E. , Yilmazer, D. , de Vos, R. A. , Jansen, E. N. , & Bohl, J. (1996). Pattern of brain destruction in Parkinson's and Alzheimer's diseases. Journal of Neural Transmission (Vienna), 103(4), 455–490. 10.1007/BF01276421 9617789

[brb31822-bib-0006] Burghart Medical Technology . Hamburg, Germany. Retrieved from: http://www.burghart.net

[brb31822-bib-0007] Cao, Y. , Fang, X. , Ottosson, J. , Naslund, E. , & Stenberg, E. (2019). A comparative study of machine learning algorithms in predicting severe complications after bariatric surgery. Journal of Clinical Medicine, 8(5), 668 10.3390/jcm8050668 PMC6571760 31083643

[brb31822-bib-0008] Conti, M. Z. , Vicini‐Chilovi, B. , Riva, M. , Zanetti, M. , Liberini, P. , Padovani, A. , & Rozzini, L. (2013). Odor identification deficit predicts clinical conversion from mild cognitive impairment to dementia due to Alzheimer's disease. Archives of Clinical Neuropsychology, 28(5), 391–399. 10.1093/arclin/act032 23669447

[brb31822-bib-0009] Devanand, D. P. , Lee, S. , Manly, J. , Andrews, H. , Schupf, N. , Doty, R. L. , … Mayeux, R. (2015). Olfactory deficits predict cognitive decline and Alzheimer dementia in an urban community. Neurology, 84(2), 182–189. 10.1212/WNL.0000000000001132 25471394 PMC4336090

[brb31822-bib-0010] Devanand, D. P. , Liu, X. , Tabert, M. H. , Pradhaban, G. , Cuasay, K. , Bell, K. , … Pelton, G. H. (2008). Combining early markers strongly predicts conversion from mild cognitive impairment to Alzheimer's disease. Biological Psychiatry, 64(10), 871–879. 10.1016/j.biopsych.2008.06.020 18723162 PMC2613777

[brb31822-bib-0011] Ding, D. , Zhao, Q. , Guo, Q. , Meng, H. , Wang, B. , Luo, J. , … Hong, Z. (2015). Prevalence of mild cognitive impairment in an urban community in China: A cross‐sectional analysis of the Shanghai Aging Study. Alzheimer's & Dementia: The Journal of the Alzheimer's Association, 11(3), 300–309. e2. 10.1016/j.jalz.2013.11.002 24613707

[brb31822-bib-0012] Ding, D. , Zhao, Q. H. , Guo, Q. H. , Meng, H. J. , Wang, B. , Yu, P. M. , … Hong, Z. (2014). The Shanghai aging study: Study design, baseline characteristics, and prevalence of dementia. Neuroepidemiology, 43(2), 114–122. 10.1159/000366163 25376362

[brb31822-bib-0013] Eaton, W. W. , Smith, C. , Ybarra, M. , Muntaner, C. , & Tien, A. (2004). Center for Epidemiologic Studies Depression Scale: Review and revision (CESD and CESD‐R) In Maruish M. E. (Ed.), The use of psychological testing for treatment planning and outcomes assessment: Instruments for adults (pp. 363–377). New York: Taylor & Francis Group.

[brb31822-bib-0014] Fancellu, G. , Chand, K. , Tomas, D. , Orlandini, E. , Piemontese, L. , Silva, D. F. , … Santos, M. A. (2020). Novel tacrine‐benzofuran hybrids as potential multi‐target drug candidates for the treatment of Alzheimer's Disease. Journal of Enzyme Inhibition and Medicinal Chemistry, 35(1), 211–226. 10.1080/14756366.2019.1689237 31760822 PMC7567501

[brb31822-bib-0015] Hartemink, A. J. (2001). Principled computational methods for the validation discovery of genetic regulatory networks. Cambridge, MA, United States: Massachusetts Institute of Technology.

[brb31822-bib-0016] Hawkes, C. H. , Shephard, B. C. , & Daniel, S. E. (1997). Olfactory dysfunction in Parkinson's disease. Journal of Neurology, Neurosurgery & Psychiatry, 62(5), 436–446. 10.1136/jnnp.62.5.436 PMC486843 9153598

[brb31822-bib-0017] James, G. , Witten, D. , Hastie, T. , & Tibshirani, R. (2013). An introduction to statistical learning, Vol. 112 New York, NY: Springer.

[brb31822-bib-0018] Kammerdiner, A. R. , Gupal, A. M. , & Pardalos, P. M. (2007). Application of Bayesian networks and data mining to biomedical problems. AIP Conference Proceedings, 953:132–+.

[brb31822-bib-0019] Karhausen, L. R. (1987). On the logic of causal inference. American Journal of Epidemiology, 126(3), 556–557. 3618587 10.1093/oxfordjournals.aje.a114690

[brb31822-bib-0020] Khanna, S. , Domingo‐Fernandez, D. , Iyappan, A. , Emon, M. A. , Hofmann‐Apitius, M. , & Frohlich, H. (2018). Using multi‐scale genetic, neuroimaging and clinical data for predicting Alzheimer's disease and reconstruction of relevant biological mechanisms. Scientific Reports, 8(1), 1–13. 10.1038/s41598-018-29433-3 30042519 PMC6057884

[brb31822-bib-0021] Koss, E. (1986). Olfactory dysfunction in Alzheimer's disease. Developmental Neuropsychology, 2(2), 89–99. 10.1080/87565648609540332

[brb31822-bib-0022] Lautrup, S. , Sinclair, D. A. , Mattson, M. P. , & Fang, E. F. (2019). NAD(+) in brain aging and neurodegenerative disorders. Cell Metabolism, 30(4), 630–655. 10.1016/j.cmet.2019.09.001 31577933 PMC6787556

[brb31822-bib-0023] Li, J. , Shi, J. J. , & Satz, D. (2008). Modeling and analysis of disease and risk factors through learning Bayesian networks from observational data. Quality and Reliability Engineering International, 24(3), 291–302. 10.1002/qre.893

[brb31822-bib-0024] Liang, X. N. , Ding, D. , Zhao, Q. H. , Guo, Q. H. , Luo, J. F. , Hong, Z. (2016). Association between olfactory identification and cognitive function in community‐dwelling elderly: The Shanghai aging study. BMC Neurology, 16(1), 199 10.1186/s12883-016-0725-x 27765032 PMC5073423

[brb31822-bib-0025] Liang, X. , Ding, D. , Zhao, Q. , Wu, W. , Xiao, Z. , Luo, J. , & Hong, Z. (2020). Inability to smell peppermint is related to cognitive decline: A prospective community‐based study. Neuroepidemiology, 54(3), 258–264. 10.1159/000505485 31935728

[brb31822-bib-0026] Lucas, P. J. (2007). Biomedical applications of Bayesian networks In Advances in probabilistic graphical models, Vol. 213 (pp. 333–358). Berlin, Heidelberg: Springer.

[brb31822-bib-0027] Mandrekar, J. N. (2010). Receiver operating characteristic curve in diagnostic test assessment. Journal of Thoracic Oncology, 5(9), 1315–1316. 10.1097/JTO.0b013e3181ec173d 20736804

[brb31822-bib-0028] Mani, S. , Valtorta, M. , & McDermott, S. (2005). Building Bayesian network models in medicine: The MENTOR experience. Applied Intelligence, 22(2), 93–108. 10.1007/s10489-005-5599-3

[brb31822-bib-0029] Marzban, C. (2004). The ROC curve and the area under it as performance measures. Weather Forecast, 19(6), 1106–1114. 10.1175/825.1

[brb31822-bib-0030] Mestizo‐Gutiérrez, S. L. , Jácome‐Delgado, J. A. , Rosales‐Morales, V. Y. , Cruz‐Ramírez, N. , & Aranda‐Abreu, G. E. (2019). A Bayesian Network Model for the Parkinson’s Disease: A study of gene expression levels In Current trends in Semantic Web Technologies: Theory and practice (pp. 153–186). Cham, Switzerland: Springer.

[brb31822-bib-0031] Moffa, G. , Catone, G. , Kuipers, J. , Kuipers, E. , Freeman, D. , Marwaha, S. , … Bebbington, P. (2017). Using directed acyclic graphs in epidemiological research in psychosis: An analysis of the role of bullying in psychosis. Schizophrenia Bulletin, 43(6), 1273–1279. 10.1093/schbul/sbx013 28521005 PMC5737513

[brb31822-bib-0032] Nguefack‐Tsague, G. (2011). Using bayesian networks to model hierarchical relationships in epidemiological studies. Epidemiology and Health, 33, e2011006 10.4178/epih/e2011006 21779534 PMC3132659

[brb31822-bib-0033] Pearl, J. (2014). Probabilistic reasoning in intelligent systems: Networks of plausible inference. San Francisco, CA: Elsevier.

[brb31822-bib-0034] Peng, C. , Trojanowski, J. Q. , & Lee, V. M. (2020). Protein transmission in neurodegenerative disease. Nature Reviews. Neurology, 16(4), 199–212. 10.1038/s41582-020-0333-7 32203399 PMC9242841

[brb31822-bib-0035] Prince, M. , Comas‐Herrera, A. , Knapp, M. , Guerchet, M. , & Karagiannidou, M. (2016). World Alzheimer report 2016: Improving healthcare for people living with dementia: Coverage, quality and costs now and in the future. London, UK: Alzheimer’s Disease International (ADI).

[brb31822-bib-0036] Rahayel, S. , Frasnelli, J. , & Joubert, S. (2012). The effect of Alzheimer's disease and Parkinson's disease on olfaction: A meta‐analysis. Behavioral Brain Research, 231(1), 60–74. 10.1016/j.bbr.2012.02.047 22414849

[brb31822-bib-0037] Ripley, B. D. (2002). Modern applied statistics with S. New York, NY: Springer.

[brb31822-bib-0038] Roalf, D. R. , Moberg, M. J. , Turetsky, B. I. , Brennan, L. , Kabadi, S. , Wolk, D. A. , & Moberg, P. J. (2017). A quantitative meta‐analysis of olfactory dysfunction in mild cognitive impairment. Journal of Neurology, Neurosurgery and Psychiatry, 88(3), 226–232. 10.1136/jnnp-2016-314638 PMC5350628 28039318

[brb31822-bib-0039] Roberts, R. O. , Christianson, T. J. H. , Kremers, W. K. , Mielke, M. M. , Machulda, M. M. , Vassilaki, M. , … Petersen, R. C. (2016). Association between olfactory dysfunction and amnestic mild cognitive impairment and Alzheimer disease dementia. JAMA Neurology, 73(1), 93–101. 10.1001/jamaneurol.2015.2952 26569387 PMC4710557

[brb31822-bib-0040] Sachs, K. , Perez, O. , Pe'er, D. , Lauffenburger, D. A. , & Nolan, G. P. (2005). Causal protein‐signaling networks derived from multiparameter single‐cell data. Science, 308(5721), 523–529. 10.1126/science.1105809 15845847

[brb31822-bib-0041] Schofield, P. W. , Finnie, S. , & Yong, Y. M. (2014). The role of olfactory challenge tests in incipient dementia and clinical trial design. Current Neurology and Neuroscience Reports, 14(9), 479 10.1007/s11910-014-0479-z 25053174

[brb31822-bib-0042] Scutari, M. , & Denis, J.‐B. (2014). Bayesian networks: With examples in R. Boca Raton, FL: Chapman and Hall/CRC.

[brb31822-bib-0043] Serby, M. , Corwin, J. , Conrad, P. , & Rotrosen, J. (1985). Olfactory dysfunction in Alzheimer's disease and Parkinson's disease. American Journal of Psychiatry, 142(6), 781–782. 10.1176/ajp.142.6.781-a 4003606

[brb31822-bib-0044] Smirnov, D. A. , Morley, M. , Shin, E. , Spielman, R. S. , & Cheung, V. G. (2009). Genetic analysis of radiation‐induced changes in human gene expression. Nature, 459(7246), 587–591. 10.1038/nature07940 19349959 PMC3005325

[brb31822-bib-0045] Stanciu, I. , Larsson, M. , Nordin, S. , Adolfsson, R. , Nilsson, L. G. , & Olofsson, J. K. (2014). Olfactory impairment and subjective olfactory complaints independently predict conversion to dementia: A longitudinal, population‐based study. Journal of the International Neuropsychological Society, 20(2), 209–217. 10.1017/S1355617713001409 24451436

[brb31822-bib-0046] Sun, G. H. , Raji, C. A. , MacEachern, M. P. , & Burke, J. F. (2012). Olfactory identification testing as a predictor of the development of Alzheimer's dementia: A systematic review. Laryngoscope, 122(7), 1455–1462. 10.1002/lary.23365 22552846

[brb31822-bib-0047] Susser, M. (1991). What is a cause and how do we know one ‐ a grammar for pragmatic epidemiology. American Journal of Epidemiology, 133(7), 635–648. 10.1093/oxfordjournals.aje.a115939 2018019

[brb31822-bib-0048] Wehling, E. , Nordin, S. , Espeseth, T. , Reinvang, I. , & Lundervold, A. J. (2011). Unawareness of olfactory dysfunction and its association with cognitive functioning in middle aged and old adults. Archives of Clinical Neuropsychology, 26(3), 260–269. 10.1093/arclin/acr019 21474482

[brb31822-bib-0049] Wolfensberger, M. (2000). Sniffin'Sticks: A new olfactory test battery. Acta oto‐laryngologica, 120(2), 303–306. 10.1080/000164800750001134 11603794

