# Supplementary material for: Can dementia be predicted using olfactory identification test in the elderly? A Bayesian network analysis
Source: Brain Behav. 2020 Aug 31;10(11):e01822. doi: 10.1002/brb3.1822 (PMC7667339; doi:10.1002/brb3.1822)
Supplement: Supplementary file 1 — Supinfo [file BRB3-10-e01822-s001.docx]

**Supplemental materials**

Table S1. Coefficients of the variables selected by bidirectional stepwise method in the multivariable logistic regression analysis

| Variable | β | SE | P-value |
| --- | --- | --- | --- |
| Age | 0.155 | 0.027 | 0.000 |
| Weight | -0.031 | 0.015 | 0.036 |
| Education | -0.081 | 0.035 | 0.020 |
| Depression | 0.708 | 0.379 | 0.061 |
| Stroke | 0.648 | 0.377 | 0.086 |
| APOEe4 | 0.839 | 0.369 | 0.023 |
| Leather | -0.514 | 0.316 | 0.104 |
| Peppermint | -1.157 | 0.388 | 0.003 |
| Banana | -0.647 | 0.308 | 0.036 |
| Lemon | 0.524 | 0.317 | 0.098 |
| Pineapple | 0.879 | 0.359 | 0.014 |
| Rose | -0.501 | 0.310 | 0.107 |
| MMSE | -0.413 | 0.079 | 0.000 |

SE, standard error; APOE, Apolipoprotein; MMSE, Mini-mental State Examination

Table S2. Performance of discrete Bayesian networks (DBN)

| Dependencies of incident dementia in the DBN | Training data | | | | | | Validation data | | | | | |
| --- | --- | --- | --- | --- | --- | --- | --- | --- | --- | --- | --- | --- |
|  | Sensitivity | Specificity | Accuracy | AUC | 95% CI of AUC | | Sensitivity | Specificity | Accuracy | AUC | 95% CI of AUC | |
|  |  |  |  |  | Lower | Upper |  |  |  |  | Lower | Upper |
| Age+OIS+Orange | 1.000 | 0.806 | 0.821 | 0.937 | 0.918 | 0.956 | 0.556 | 0.778 | 0.756 | 0.653 | 0.525 | 0.781 |
| Age+OIS+Leather | 1.000 | 0.811 | 0.825 | 0.947 | 0.930 | 0.964 | 0.421 | 0.756 | 0.722 | 0.560 | 0.440 | 0.679 |
| Age+OIS+Cinnamon | 1.000 | 0.826 | 0.839 | 0.941 | 0.924 | 0.959 | 0.316 | 0.917 | 0.856 | 0.616 | 0.487 | 0.746 |
| Age+OIS+Peppermint | 1.000 | 0.775 | 0.792 | 0.933 | 0.913 | 0.954 | 0.579 | 0.768 | 0.749 | 0.663 | 0.534 | 0.793 |
| Age+OIS+Banana | 1.000 | 0.798 | 0.813 | 0.938 | 0.919 | 0.956 | 0.529 | 0.790 | 0.765 | 0.659 | 0.521 | 0.797 |
| Age+OIS+Lemon | 1.000 | 0.813 | 0.827 | 0.935 | 0.916 | 0.954 | 0.529 | 0.770 | 0.747 | 0.627 | 0.501 | 0.753 |
| Age+OIS+Liquorice | 1.000 | 0.832 | 0.844 | 0.944 | 0.927 | 0.961 | 0.471 | 0.848 | 0.812 | 0.648 | 0.514 | 0.783 |
| Age+OIS+Coffee | 1.000 | 0.765 | 0.782 | 0.924 | 0.903 | 0.946 | 0.526 | 0.732 | 0.711 | 0.617 | 0.486 | 0.748 |
| Age+OIS+Cloves | 1.000 | 0.801 | 0.815 | 0.933 | 0.914 | 0.953 | 0.556 | 0.776 | 0.755 | 0.671 | 0.538 | 0.804 |
| Age+OIS+Pineapple | 1.000 | 0.803 | 0.818 | 0.943 | 0.925 | 0.961 | 0.474 | 0.814 | 0.778 | 0.623 | 0.496 | 0.751 |
| Age+OIS+Rose | 1.000 | 0.819 | 0.832 | 0.937 | 0.919 | 0.955 | 0.611 | 0.748 | 0.735 | 0.698 | 0.563 | 0.833 |
| Age+OIS+Fish | 1.000 | 0.789 | 0.805 | 0.928 | 0.908 | 0.948 | 0.500 | 0.766 | 0.741 | 0.628 | 0.491 | 0.764 |
| Age+Edu+Orange | 1.000 | 0.806 | 0.821 | 0.946 | 0.929 | 0.963 | 0.824 | 0.752 | 0.759 | 0.784 | 0.677 | 0.891 |
| Age+Edu+Leather | 1.000 | 0.848 | 0.859 | 0.953 | 0.937 | 0.968 | 0.611 | 0.776 | 0.760 | 0.698 | 0.571 | 0.824 |
| Age+Edu+Cinnamon | 1.000 | 0.829 | 0.842 | 0.953 | 0.938 | 0.969 | 0.579 | 0.773 | 0.753 | 0.678 | 0.555 | 0.801 |
| Age+Edu+Peppermint | 1.000 | 0.808 | 0.822 | 0.957 | 0.942 | 0.973 | 0.722 | 0.768 | 0.764 | 0.747 | 0.629 | 0.866 |
| Age+Edu+Banana | 1.000 | 0.843 | 0.855 | 0.955 | 0.940 | 0.970 | 0.684 | 0.799 | 0.787 | 0.757 | 0.638 | 0.876 |
| Age+Edu+Lemon | 1.000 | 0.853 | 0.864 | 0.958 | 0.944 | 0.973 | 0.556 | 0.798 | 0.773 | 0.685 | 0.556 | 0.815 |
| Age+Edu+Liquorice | 1.000 | 0.830 | 0.843 | 0.951 | 0.935 | 0.967 | 0.500 | 0.765 | 0.739 | 0.635 | 0.508 | 0.762 |
| Age+Edu+Coffee | 1.000 | 0.781 | 0.797 | 0.941 | 0.922 | 0.960 | 0.684 | 0.727 | 0.723 | 0.738 | 0.612 | 0.864 |
| Age+Edu+Cloves | 1.000 | 0.849 | 0.860 | 0.957 | 0.943 | 0.972 | 0.526 | 0.783 | 0.757 | 0.653 | 0.530 | 0.776 |
| Age+Edu+Pineapple | 1.000 | 0.830 | 0.843 | 0.954 | 0.939 | 0.970 | 0.684 | 0.795 | 0.784 | 0.728 | 0.614 | 0.842 |
| Age+Edu+Rose | 1.000 | 0.842 | 0.854 | 0.958 | 0.944 | 0.973 | 0.611 | 0.807 | 0.788 | 0.697 | 0.574 | 0.820 |
| Age+Edu+Fish | 1.000 | 0.840 | 0.852 | 0.956 | 0.942 | 0.971 | 0.684 | 0.820 | 0.806 | 0.747 | 0.631 | 0.863 |
| Age+APOEe4+Orange | 0.821 | 0.744 | 0.749 | 0.861 | 0.822 | 0.900 | 0.789 | 0.689 | 0.699 | 0.728 | 0.606 | 0.850 |
| Age+APOEe4+Leather | 0.875 | 0.731 | 0.741 | 0.868 | 0.834 | 0.903 | 0.737 | 0.700 | 0.704 | 0.746 | 0.615 | 0.876 |
| Age+APOEe4+Cinnamon | 0.875 | 0.712 | 0.724 | 0.871 | 0.836 | 0.907 | 0.842 | 0.653 | 0.672 | 0.778 | 0.671 | 0.885 |
| Age+APOEe4+Peppermint | 0.839 | 0.729 | 0.737 | 0.876 | 0.839 | 0.912 | 0.789 | 0.671 | 0.683 | 0.734 | 0.610 | 0.858 |
| Age+APOEe4+Banana | 0.946 | 0.662 | 0.683 | 0.873 | 0.838 | 0.908 | 0.789 | 0.724 | 0.730 | 0.761 | 0.641 | 0.880 |
| Age+APOEe4+Lemon | 0.911 | 0.691 | 0.707 | 0.870 | 0.835 | 0.904 | 0.684 | 0.735 | 0.730 | 0.720 | 0.604 | 0.837 |
| Age+APOEe4+Liquorice | 0.911 | 0.691 | 0.707 | 0.872 | 0.838 | 0.906 | 0.684 | 0.729 | 0.725 | 0.722 | 0.589 | 0.854 |
| Age+APOEe4+Coffee | 0.857 | 0.745 | 0.753 | 0.873 | 0.836 | 0.910 | 0.789 | 0.671 | 0.683 | 0.754 | 0.635 | 0.873 |
| Age+APOEe4+Cloves | 0.839 | 0.739 | 0.747 | 0.865 | 0.828 | 0.902 | 0.737 | 0.741 | 0.741 | 0.771 | 0.648 | 0.895 |
| Age+APOEe4+Pineapple | 0.821 | 0.745 | 0.751 | 0.863 | 0.825 | 0.900 | 0.789 | 0.712 | 0.720 | 0.755 | 0.645 | 0.865 |
| Age+APOEe4+Rose | 0.893 | 0.687 | 0.702 | 0.869 | 0.832 | 0.905 | 0.737 | 0.712 | 0.714 | 0.741 | 0.609 | 0.872 |
| Age+APOEe4+Fish | 0.893 | 0.687 | 0.702 | 0.873 | 0.837 | 0.908 | 0.842 | 0.624 | 0.646 | 0.777 | 0.669 | 0.884 |
| Age+Edu+MMSE+Orange | 1.000 | 0.966 | 0.968 | 0.994 | 0.991 | 0.998 | 0.667 | 0.919 | 0.884 | 0.783 | 0.641 | 0.924 |
| Age+Edu+MMSE+Leather | 1.000 | 0.974 | 0.976 | 0.996 | 0.994 | 0.999 | 0.545 | 0.930 | 0.878 | 0.730 | 0.575 | 0.886 |
| Age+Edu+MMSE+Cinnamon | 1.000 | 0.972 | 0.974 | 0.995 | 0.992 | 0.998 | 0.667 | 0.909 | 0.880 | 0.787 | 0.619 | 0.955 |
| Age+Edu+MMSE+Peppermint | 1.000 | 0.969 | 0.971 | 0.995 | 0.992 | 0.998 | 0.538 | 0.906 | 0.857 | 0.720 | 0.576 | 0.864 |
| Age+Edu+MMSE+Banana | 1.000 | 0.973 | 0.975 | 0.996 | 0.994 | 0.999 | 0.364 | 0.904 | 0.833 | 0.628 | 0.478 | 0.777 |
| Age+Edu+MMSE+Lemon | 1.000 | 0.973 | 0.975 | 0.997 | 0.994 | 0.999 | 0.636 | 0.902 | 0.861 | 0.757 | 0.599 | 0.915 |
| Age+Edu+MMSE+Liquorice | 1.000 | 0.972 | 0.974 | 0.996 | 0.993 | 0.999 | 0.462 | 0.921 | 0.842 | 0.687 | 0.543 | 0.831 |
| Age+Edu+MMSE+Coffee | 1.000 | 0.960 | 0.963 | 0.993 | 0.989 | 0.997 | 0.500 | 0.895 | 0.840 | 0.700 | 0.558 | 0.841 |
| Age+Edu+MMSE+Cloves | 1.000 | 0.972 | 0.974 | 0.996 | 0.993 | 0.999 | 0.462 | 0.850 | 0.781 | 0.656 | 0.506 | 0.805 |
| Age+Edu+MMSE+Pineapple | 1.000 | 0.970 | 0.972 | 0.995 | 0.992 | 0.998 | 0.500 | 0.913 | 0.843 | 0.705 | 0.565 | 0.845 |
| Age+Edu+MMSE+Rose | 1.000 | 0.974 | 0.976 | 0.997 | 0.995 | 0.999 | 0.545 | 0.896 | 0.846 | 0.719 | 0.560 | 0.878 |
| Age+Edu+MMSE+Fish | 1.000 | 0.977 | 0.979 | 0.997 | 0.995 | 0.999 | 0.462 | 0.906 | 0.847 | 0.691 | 0.543 | 0.839 |


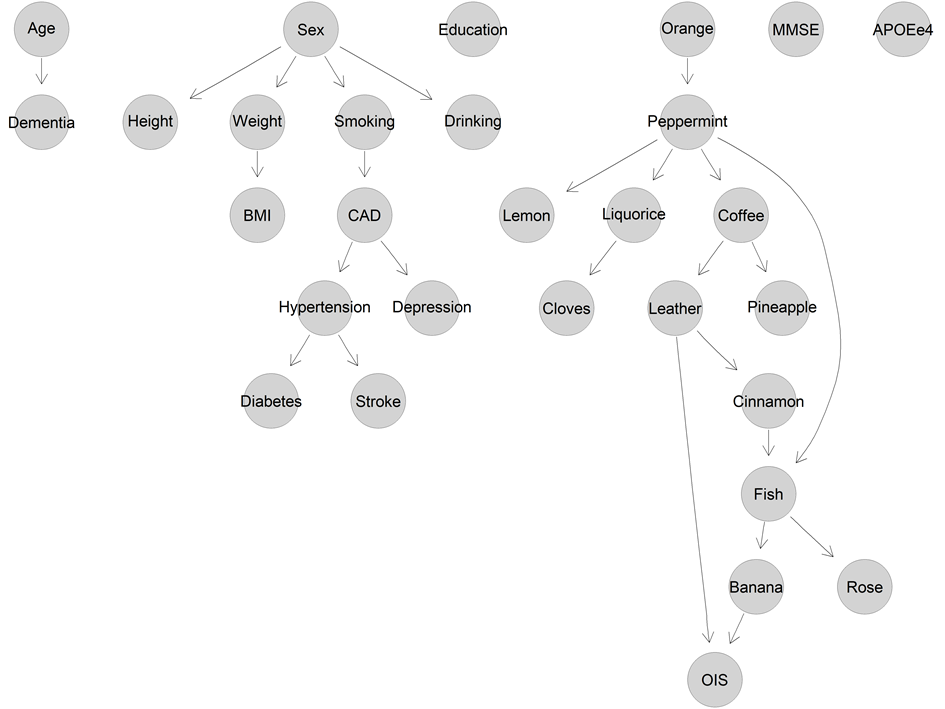


Figure S1. Structure of the initially learnt discrete Bayesian network for predicting incident dementia using the baseline variables
